# Sustainable effect of individualised sun protection advice on sun protection behaviour: a 10-year follow-up of a randomised controlled study in primary care

**DOI:** 10.3399/bjgpopen19X101653

**Published:** 2019-07-24

**Authors:** Henrik Hedevik, Ghassan Guorgis, Chris D Anderson, Magnus Falk

**Affiliations:** 1 Statistician, Department of Medical and Health Sciences, Division of Physiotherapy, Linköping University, Linköping, Sweden; 2 Doctoral Student, Department of Medical and Health Sciences, Division of Community Medicine, Primary Care, Linköping University, Linköping, Sweden; 3 Professor, Department of Clinical and Experimental Medicine, Division of Dermatology, Linköping University, Linköping, Sweden; 4 Associate Professor, Department of Medical and Health Sciences, Division of Community Medicine, Primary Care, Linköping University, Linköping, Sweden

**Keywords:** Primary prevention, Primary Health Care, Questionnaire, Randomized controlled trial, Skin cancers, Ultraviolet exposure

## Abstract

**Background:**

In the light of increasing skin cancer incidences worldwide, preventive measures to promote sun protection in individuals with risky sun habits have continued relevance and importance.

**Aim:**

To report the long-term effect of individualised sun protection advice given in primary health care (PHC), on sun habits and sun protection behaviour.

**Design & setting:**

In 2005, 309 PHC patients were enrolled in a randomised controlled study performed in a Swedish PHC setting.

**Method:**

At baseline, the study participants completed a Likert scale-based questionnaire, mapping sun habits, propensity to increase sun protection, and attitudes towards sun exposure, followed by randomisation into three intervention groups, all receiving individualised sun protection advice: in Group 1 (*n* = 116) by means of a letter, and in Group 2 (*n* = 97) and 3 (*n* = 96) communicated personally by a GP. In Group 3, participants also underwent a skin ultraviolet-sensitivity phototest, with adjusted sun protection advice based on the result. A repeated questionnaire was administered after 3 and 10 years.

**Results:**

Statistically significant declines were observed in all groups for sun exposure mean scores over time. When using a cumulative score, according to the Sun Exposure and Protection Index (SEPI), significantly greater decrease in SEPI mean score was observed in Groups 2 and 3 (GP), compared to Group 1 (letter); *P*<0.01. The addition of a phototest did not enhance the effect of the intervention.

**Conclusion:**

Individualised sun protection advice mediated verbally by the GP can lead to sustained improvement of sun protective behaviour.

## How this fits in

Individualised sun protection advice to reduce ultraviolet (UV) exposure has been shown in systematic reviews to have effect. No studies, however, have followed the participants for more than 1 year after an intervention, and thus there is a lack of knowledge on long-term sustainability of the effect of behavioural interventions to increase sun protection. In the present study, performed in primary care, 10-year follow-up of the participants showed persistent, statistically significant increase of sun protection after advice when mediated personally by a GP.

## Introduction

Increasing skin cancer incidence is associated with substantial patient suffering and healthcare costs worldwide,^1–5^ emphasising the necessity of preventive measures directed towards the disease. The role and effectiveness of interventions to promote sun avoidance and protection in order to prevent skin cancer have been studied and debated during recent decades. Studied interventions range from local educational or informational efforts directed at defined target groups to broad, national government-initiated campaigns, and include a variety of methodological and informational approaches (ranging from brief, written information sheets to personalised face-to-face mediated advice). Interventions have been reported to have had varying success^6–9^ but, based on systematic reviews, there is a consensus that educational measures to increase sun protection are both effective and worthwhile, at least when directed at younger individuals who are at the highest risk of establishing a future lifetime risk for skin cancer.^7–9^ A complicating factor in this respect is the difficulty of actually demonstrating not only the effect of interventions to reduce UV exposure, but also, in the longer term, the effect on skin cancer incidence.^6,8^ Instead, present knowledge relies on the reasoning that if sun protection advice is proven to lead to increased sun protection, and increased sun protection per se is known to be associated with a reduction in risk for developing skin cancer,^10–12^ it is likely that measures efficient in promoting sun protection will also reduce the likelihood of developing skin cancer in the future. In most studies, however, follow-up intervals are short,^6–9^ making conclusions on sustainability of any observed behavioural change difficult to draw. Additionally, since the main negative effects of UV radiation (such as skin cancer) derive from long-term exposure, any behaviour change in a favourable direction would need to be maintained over a longer period of time to have effect.

Another issue is the balance between beneficial and harmful effects of UV radiation. Lately, increasing attention has been directed towards vitamin-D deficiency, and since UV exposure (in moderation) may also have other beneficial effects, with regard to the individual’s whole health perspective, not all individuals would necessarily gain from reducing sun exposure.^13–15^ Variations in intensity of UV radiation, according to geographic location and latitude, and fluctuation between seasons need to be taken into account. Therefore, if undertaken by healthcare providers, there is a reliance on the performing physician to balance the content of the advice given, and to direct it towards those most likely to gain from it. This demands adequate consideration of the patient’s integrated health state and history, a task often undertaken by the GP.

The aim of the present study was to investigate the long-term (10-year) effect of individualised sun protection advice, mediated by the GP, on sun exposure habits and sun protection behaviour.

## Method

**Figure 1.**
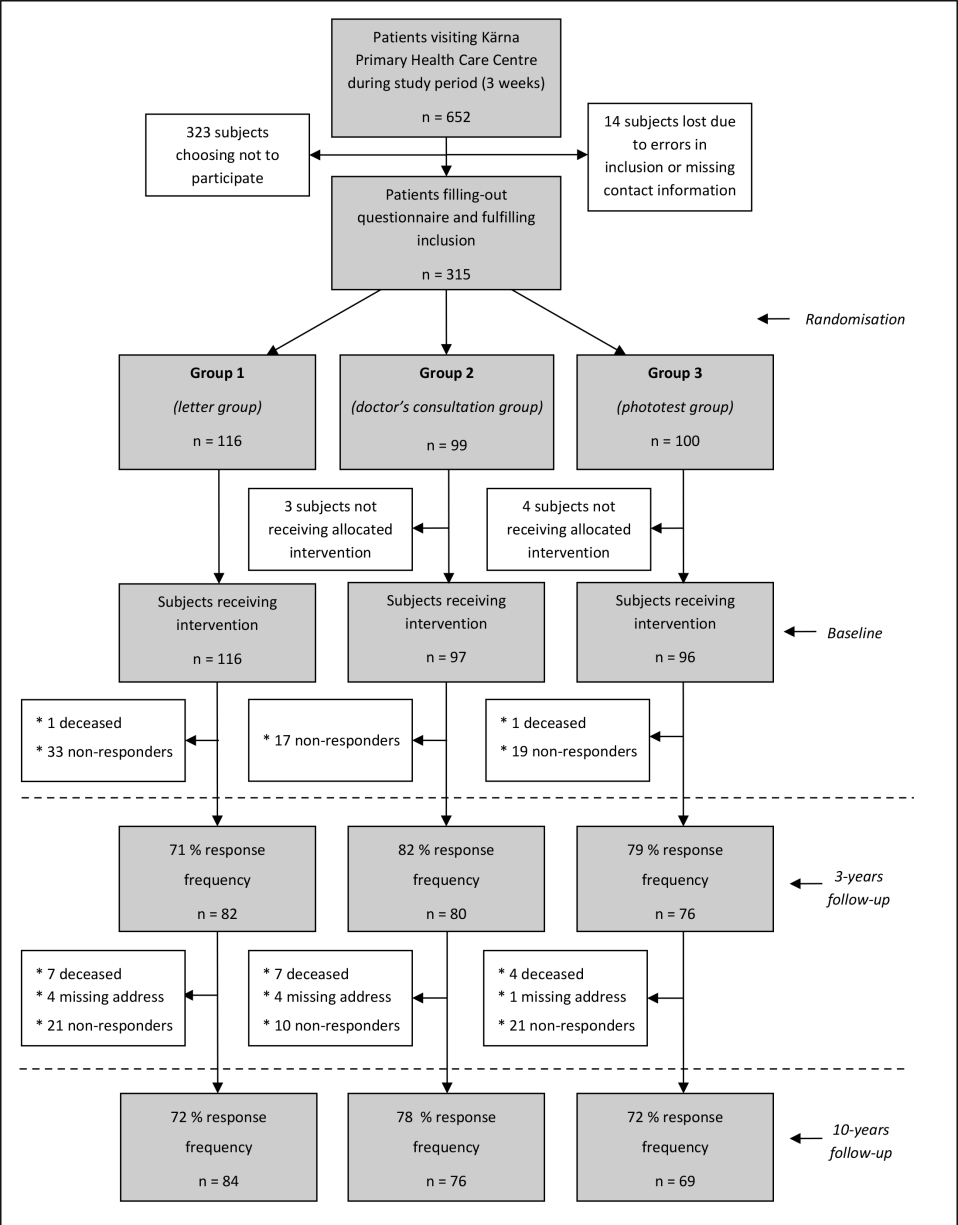
Distribution of study participants in the three intervention groups, and response frequencies at baseline, at 3-year, and at 10-year follow-up. The percentages given at 3 and 10 years describe the proportional response rate with regard to baseline

The study was launched in 2005, in the form of a randomised controlled trial (Figure 1).^16,17^ During 3 weeks in the month of February (hereafter referred to as ‘baseline’), independently of the purpose of their visit, all patients aged ≥18 years registering in the reception at a PHC centre in the city of Linköping, Sweden, received written information on the study, together with a request to participate, a consent form, and a questionnaire. At study outset, the Linköping municipality had approximately 140 000 inhabitants, of which around 13 500 were allocated to the PHC centre in question. The consent form and the questionnaire could either be filled out while at the PHC centre and returned to the receptionist or placed in a response box in the waiting room, or filled out later at home and returned by post. Abnormal UV sensitivity, intake of UV-sensitising medication, and cognitive impairment were exclusion criteria. After inclusion, participants were consecutively computer-randomised, by a member of administrative staff, to one of three study groups. Individualised sun protection advice based on their questionnaire responses was given, in varying form depending on group; in Group 1 by means of a letter, with standardised comments on skin type and sun exposure habits, and a summarised risk assessment with personally-adjusted advice, and in Groups 2 and 3 mediated verbally through a personal GP consultation (approximately 20 minutes, including a whole-body inspection of nevi). In Group 3, the participants also underwent a phototest for assessment of individual UV sensitivity (Skin-tester Kit, Cosmedico-Medizintechnik-GmbH, Schwennigen, Germany). The test was applied on the palmar side of the forearm, by illumination of six 12 mm square, closely located skin areas for each field, with increasing UV dose (18, 35, 51, 63, 82, and 105 mJ/cm^2^). After 24 hours, the participants read and reported their phototest result, by counting the number of erythemas, on a specific protocol returned by mail (an assessment procedure proven reliable).^18,19^ Finally, written, adjusted sun protection advice based on the phototest was mailed back to the participants. Based on the sample sizes and outcomes of previous studies using equivalent measures,^20–23^ a sample size of *n* = 100 (including margin for possible drop-outs) was aimed for.

### The questionnaire

The questionnaire covered the following three aspects: a) sun habits and sun protection behaviour, using 5-point Likert scales (for example, never/seldom/sometimes/often/always); b) propensity to increase sun protection, based on the *Transtheoretical Model of Behaviour Change* (TTM); and c) attitudes towards sunbathing, also expressed via Likert scales. The TTM is based on classification of the individual, using grading statements, into one of five stages representing increasing propensity to change behaviour; *pre-contemplation, contemplation, preparation, action,* or *maintenance* stage,^24^ by which four behavioural items were investigated: a) giving up sunbathing, b) using clothes for sun protection, c) using sunscreens, and d) seeking the shade during midday. Stage-of-change at analysis was scored 1–5, from maintenance to pre-contemplation stage, reflecting a declining propensity to increase protection. Similarly, the previously validated Likert scale responses concerning sun habits and attitudes towards sunbathing^20,25^ were scored 1–5 in a direction corresponding to an increasingly risky behaviour or attitude (that is, a positive attitude towards sun exposure). The questions and response alternatives for the Likert-scaled questions are available from the authors on request. Demographic data collected were age, sex, educational level, skin type according to Fitzpatrick,^26^ and personal or family-related history of skin cancer.

### Sun Exposure and Protection Index (SEPI)

Eight of the questions regarding sun habits and sun protection behaviour (intentional tanning; vacation at sunny resorts; sunscreen use; using a long-sleeved shirt or sweater; using a sun hat; occasions with sunburn; time spent in midday sun; and staying in the shade) corresponded closely to the subsequently developed and validated Sun Exposure and Protection Index (SEPI) questionnaire,^27^ in which sun exposure habits are scored from 0 to 32 points, reflecting an increasing UV risk exposure. At analysis, the responses to these eight questions were, in addition to follow-up of each individual question outcome, added together in a cumulated score, to be comparable to the SEPI score.

### Follow-up

Possible effects of the intervention (given at baseline), in terms of change of self-reported sun protection behaviour and propensity to increase sun protection (primary outcome variable), and attitudes towards sunbathing (secondary outcome variable), were, as reported in previous publications, assessed at 6 months^16^ and 3 years,^17^ by a repeated postal questionnaire. At both these follow-up occasions, the participants in the two groups which had received sun protection advice verbally from the GP (Groups 2 and 3), had significantly increased their precaution with regards the sun, in contrast to the letter group (Group 1), as presented previously.^16,17^ In the present study, the questionnaire was repeated at 10 years from baseline (again by post). Additionally, as well as individual question response comparisons, assessment according to SEPI was applied to both the 3- and 10-year responses. If a questionnaire response was not received within 3 weeks, the questionnaire was posted a second time, together with a reminder.

### Statistical analyses

Change in questionnaire responses between baseline and 3 and 10 years, was assessed by linear model of longitudinal data, using restricted maximum likelihood estimation of variance components to handle the unbalanced data. Response outcomes in the four domains (sun habits [13 questions]; propensity to increase sun protection [four questions]; attitudes towards sun exposure [five questions]; and SEPI) were analysed separately. Each model included time (three levels: baseline, 3 years, and 10 years), intervention group (three levels: Groups 1, 2, and 3), and the interaction between time and intervention group as fixed factors. Based on the information criteria of the models, an unstructured method was used for estimation of the covariance parameters of the repeated effects (time). Additional contrast analyses on change between time points were produced to assess differences in change between the intervention groups. *P* values <0.05 were considered statistically significant. Šidák correction was used to control for familywise error rate in multiple comparisons. IBM SPSS Statistics for Windows (version 23.0) was used for all analyses.

## Results

Figure 1 is a cumulative flow chart of the study participants from baseline to follow-up. At 3 and 10 years, the total response rate was 77% and 74%, respectively, compared to baseline. Demographic characteristics of the study population are shown in Table 1.

**Table 1.** Demographic characteristics of the study population at baseline and at 10-year follow-up

	Baseline		10-yearfollow-up	
	*n*	(%)	*n*	(%)
**Sex**				
Female	190	(61.5)	135	(59.0)
Male	119	(38.5)	94	(41.0)
**Age at baseline, years**				
18–25	13	(4.2)	8	(3.5)
26–40	66	(21.4)	47	(20.5)
41–65	163	(52.8)	129	(56.3)
>65	67	(21.7)	45	(19.7)
**Educational level**				
Elementary school	111	(35.9)	83	(36.2)
Upper secondary school	126	(40.8)	93	(40.6)
University degree	72	(23.3)	53	(23.1)
**Skin type (Fitzpatrick’s classification)**				
High UV sensitivity (Type I–II)	99	(32.1)	74	(32.5)
Low UV sensitivity (Type III–VI)	209	(67.9)	154	(67.5)
**Previous history of skin cancer^a^**				
No skin cancer	290	(96.0)	194	(84.7)
Basal cell carcinoma (BCC)	6	(2.0)	10	(4.4)
Squamous skin carcinoma (SCC	0	(0.0)	1	(0.4)
Malignant melanoma (MM)	5	(1.7)	5	(2.2)
Skin cancer, type unknown by responder	1	(0.3)	19	(8.3)

^a^Diagnosis distribution between groups at 10 years: Group 1: 2 BCC, 2 MM, 5 type unknown. Group 2: 3 BCC, 1 SCC, 2 MM, 6 type unknown. Group 3: 5 BCC, 1 MM, 8 type unknown.

### Sun habits and sun protection behaviour

**Figure 2.**
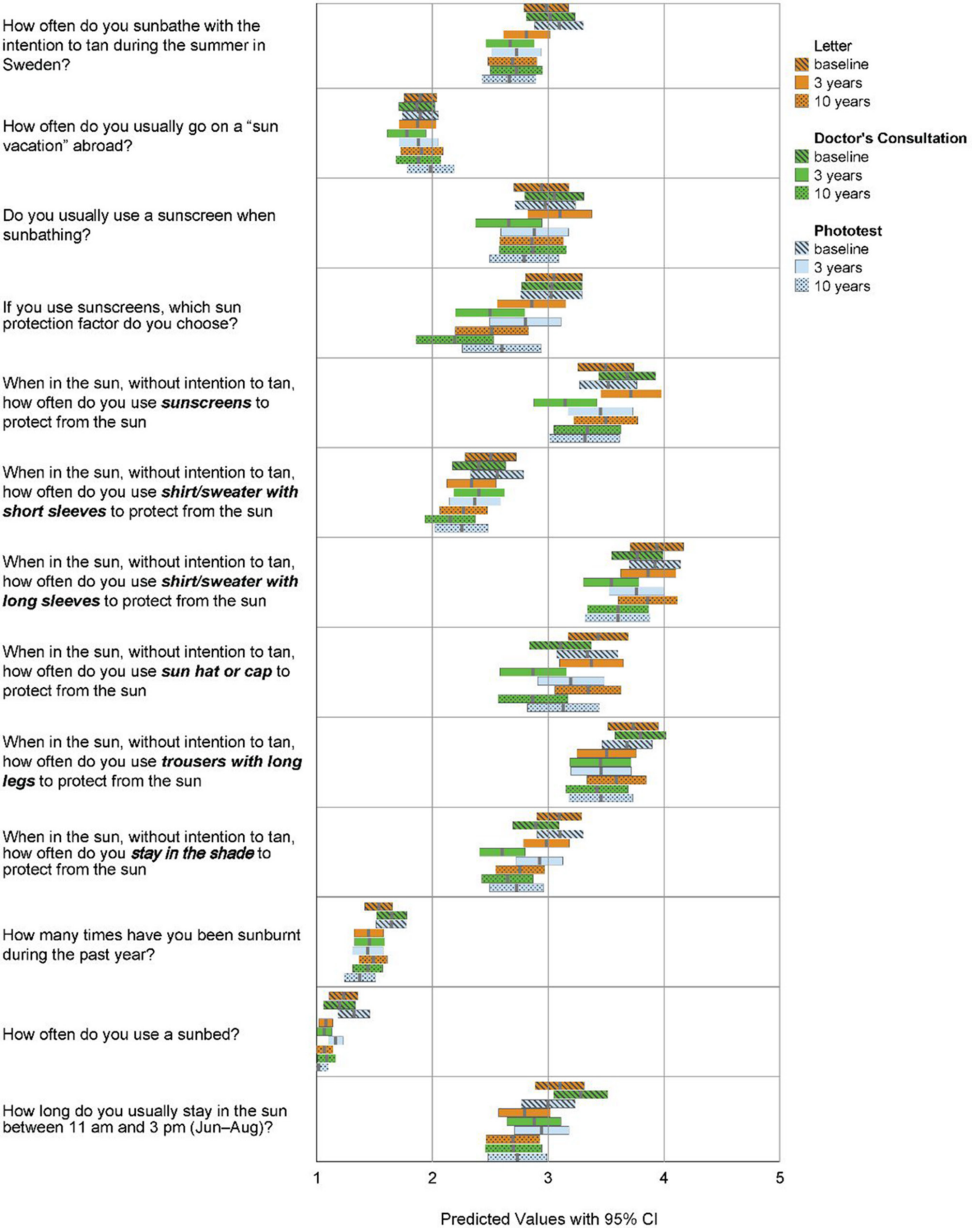
Predicted mean response values with 95% CI on each of the questions regarding sun habits and sun protection behaviour, at baseline and at 3- and 10-year follow-up, in each of the three intervention groups

Figure 2 shows the predicted mean response outcomes with 95% confidence intervals (CI), for the questions regarding sun habits and sun protection behaviour, at baseline and follow-up. For all questions, a declining mean score, corresponding to decreasing UV risk exposure, over time could be seen, independent of group. The most salient between-group differences at follow-up were seen for ‘sunscreen use’ and ‘use of clothes for protection‘, where the doctor’s consultation group (Group 2) responses were on average lower (implying less risk) compared to the letter group (Group 1). Table 2 shows the mean changes of score between the three measurement occasions, and the *P* value according to the overall effect of time, group and a combination of both. Statistically significant decreases in sun exposure score was observed within several behaviour aspects, such as intentional tanning and sunscreen use, both at 3 and 10 years compared to baseline. However, none of these changes was found to be group-dependent, rather indicating an effect of time.

**Table 2.** Mean changes of score between the three follow-up occasions (baseline, 3 years, and 10 years), regarding sun habits and sun protection behaviour, and the statistical significance according to the overall effect of time, group, and a combination of both

Model results												
	Change in estimated marginal means									Tests of fixed effects, *P* value		
	Letter			Doctor's consultation			Phototest					
	A	B	C	A	B	C	A	B	C	Time	Group	Time x Group
**Sun habits/sun protection behaviour**												
How often do you sunbathe with the intention to tan during the summer in Sweden?	-0.17	-0.12	-0.29^a^	-0.35^b^	0.06	-0.30^a^	-0.37^b^	-0.06	-0.43^b^	<0.001	0.979	0.402
How often do you usually go on a ‘sun vacation‘ abroad?	-0.02	0.04	0.01	-0.09	0.10	0.01	-0.01	0.10	0.09	0.220	0.748	0.897
Do you usually use a sunscreen when sunbathing?	0.16	-0.24	-0.08	-0.39^a^	0.21	-0.18	-0.09	-0.09	-0.18	0.125	0.787	0.032
If you use sunscreens, which sun protection factor do you choose?	-0.19	-0.34	-0.54^a^	-0.54^a^	-0.30	-0.84^b^	-0.23	-0.20	-0.43	<0.001	0.281	0.371
When in the sun, without intention to tan, how often do you use any of the following ways to protect from the sun:												
(a) sunscreens	0.22	-0.22	0.00	-0.53^b^	0.19	-0.34	-0.07	-0.14	-0.20	0.093	0.457	0.004
(b) shirt or sweater with short sleeves	-0.16	-0.07	-0.23	0.00	-0.25	-0.25	-0.19	-0.12	-0.31	0.009	0.805	0.708
(c) shirt or sweater with long sleeves	-0.08	-0.00	-0.08	-0.22	0.06	-0.17	-0.16	-0.16	-0.32	0.018	0.199	0.654
(d) sun hat or cap	-0.06	-0.03	-0.09	-0.24	-0.00	-0.24	-0.14	-0.06	-0.21	0.068	0.038	0.890
(e) trousers with long legs	-0.23	0.09	-0.14	-0.35^a^	-0.03	-0.38^a^	-0.22	-0.00	-0.23	<0.001	0.864	0.677
(f) staying in the shade	-0.11	-0.23	-0.34^a^	-0.29^c^	0.04	-0.25	-0.18	-0.20	-0.38^a^	<0.001	0.111	0.384
How many times have you been sunburnt during the past year?	-0.09	0.04	-0.05	-0.19^c^	-0.02	-0.21	-0.20^c^	-0.07	-0.27^a^	<0.001	0.907	0.291
How often do you use a sunbed?	-0.15^c^	-0.01	-0.17	-0.13	0.02	-0.11	-0.16^c^	-0.15^a^	-0.31^b^	<0.001	0.542	0.109
How long do you usually stay in the sun between 11am and 3pm (Jun–Aug)?	-0.30^c^	-0.10	-0.40^a^	-0.40^a^	-0.18	-0.58^b^	-0.06	-0.21	-0.27	<0.001	0.791	0.222
**Propensity to increase sun protection**												
Giving up sunbathing	-0.47^a^	-0.17	-0.65^b^	-0.63^b^	-0.03	-0.66^b^	-0.49^a^	-0.24	-0.76^b^	<0.001	0.510	0.909
Using clothes for sun protection	-0.22	0.04	-0.17	-0.26	0.08	-0.18	-0.29	-0.07	-0.35	0.006	0.199	0.925
Using sunscreens	-0.07	0.03	-0.05	-0.32	0.28	-0.04	-0.09	0.04	-0.05	0.131	0.557	0.658
Staying in the shade	-0.34^c^	0.76^b^	0.42^a^	-0.74^b^	0.93^b^	0.19	-0.22	0.82^b^	0.60^b^	<0.001	0.626	0.073
**Attitudes towards sun exposure**												
How do you like sunbathing?	-0.11	-0.18	-0.29^a^	-0.10	-0.00	-0.10	0.03	-0.11	-0.08	0.015	0.279	0.333
Do you think that the advantages of sunbathing outweigh the disadvantages?	-0.29^c^	-0.15	-0.44^b^	-0.29	0.14	-0.15	-0.04	-0.22	-0.26	<0.001	0.885	0.139
How extensive do you consider the health risks of sunbathing?	-0.06	-0.14	-0.20	-0.35^a^	0.19	-0.16	0.08	-0.05	-0.03	0.079	0.895	0.028
How extensive do you consider the risk for you to develop skin cancer?	-0.11	-0.02	-0.14	-0.07	0.00	-0.06	0.00	-0.19	-0.19	0.034	0.329	0.531
How important is it for you to get tanned during the summer?	-0.08	-0.11	-0.18^c^	0.01	-0.14	-0.13	-0.15	-0.13	-0.28^a^	<0.001	0.077	0.542
**SEPI score**	-0.45	-0.37	-0.82	-2.20^b^	0.15	-2.04^b^	-1.20^a^	-0.83	-2.04^b^	<0.001	0.312	0.005

^a^ *P*<0.01. ^b^ *P*<0.001. ^c^ *P*<0.05.

A = change baseline to 3 years. B = change 3 to 10 years. C = change baseline to 10 years.

A negative value indicates change towards lowered risk behaviour, increased propensity to change behaviour, and lowered risk attitude, respectively. Šidák adjustment for multiple comparisons.

### Propensity to increase sun protection

**Figure 3.**
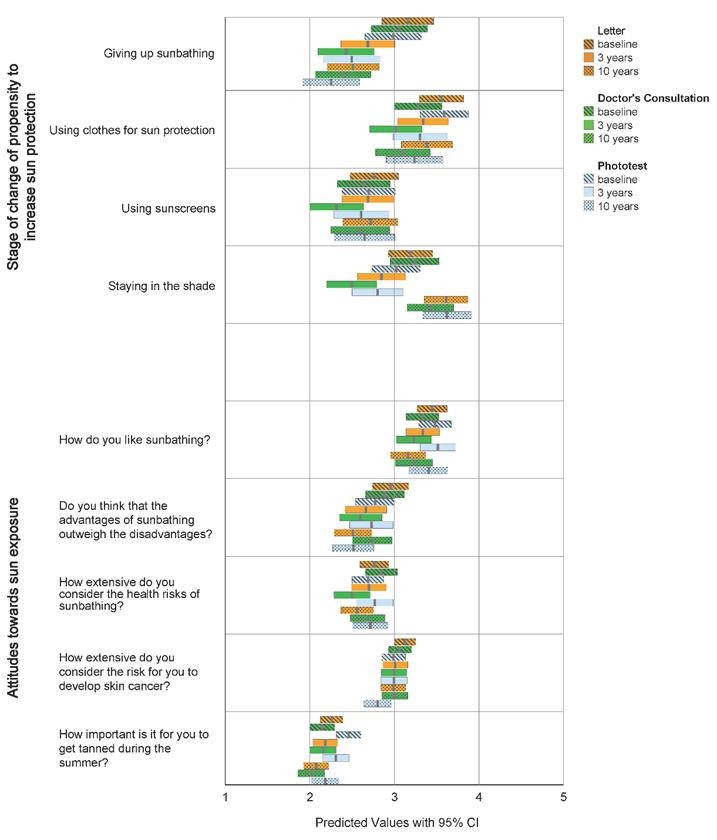
Predicted mean response values with 95% CI for each of the questions addressing stage of change of propensity to increase sun protection (A), and the questions regarding attitudes towards sun exposure (B), at baseline and at 3 and 10 years follow-up, in each of the three intervention groups

Figure 3A shows the predicted mean response outcome with its 95% CI for each of the four questions regarding readiness to increase sun protection. The time-dependent pattern of declining mean score observed in Figure 2, for sun habits, was not as obvious in this case, with the exception of the ‘giving up sunbathing‘ item. Again, however, a difference especially between the doctor’s consultation group (Group 2) and the letter group (Group 1) responses at follow-up were seen for all four questions. As seen in Table 2, all statistically significant changes of propensity-to-change score noted appeared to be dependent on time, with no significant group-dependent changes found.

### Attitudes towards sun exposure

Figure 3B shows the predicted mean response outcome with its 95% CI for the questions concerning attitudes towards sun exposure. A slight tendency towards a less positive attitude to sun exposure could be seen over time, but between-group differences were smaller. As illustrated in Table 2, observed changes in attitude were shown to be time-dependent.

### SEPI

**Figure 4.**
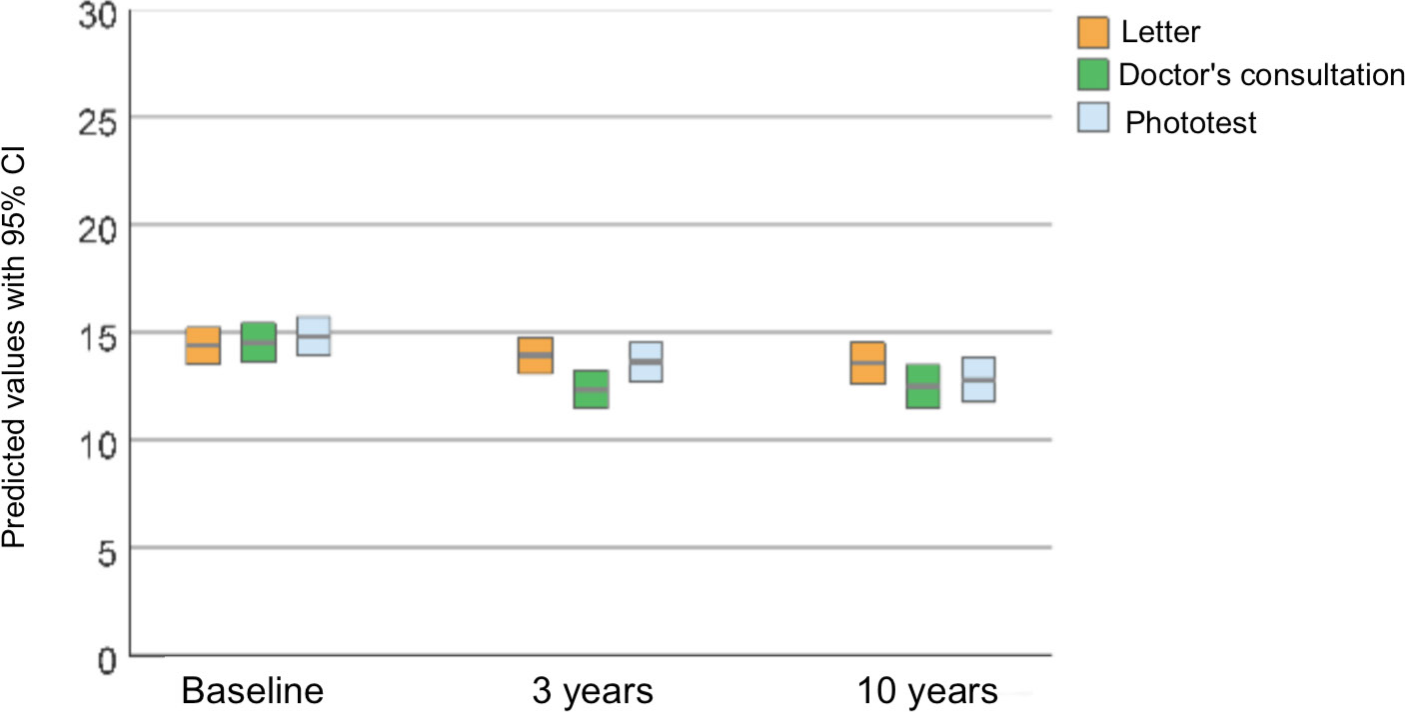
Predicted mean Sun Exposure and Protection Index score with 95% CI, at baseline and at 3- and 10-year follow-up, in each of the three intervention groups

The results of accumulated question responses added together in a comprehensive score, following the contents of SEPI, are shown in Figure 4. A greater decrease in score for Groups 2 and 3 was seen at both 3 and 10 years. Whereas no significant change in SEPI score could be detected in Group 1, the scores in Groups 2 and 3 decreased by around 2.0 in mean score (Table 2). Even after accounting for the effect of time, a significant group-dependent effect could be demonstrated.

## Discussion

### Summary

Time is a crucial factor in the prevention of diseases associated with long-term exposures, as in the case of chronic or repeated UV exposure. In this study investigating the sustainability of GP-delivered, individualised sun protection advice, a declining but detectable improvement in sun protection, over a longer perspective of time, could be demonstrated. Time per se (or, more likely, ageing) appears as the dominating factor behind this, but, importantly, individuals who received the advice personally from the doctor had significantly lower SEPI scores at 10 years than those given only written advice, suggesting this way of communicating sun protection advice to be more successful, as well as sustainable. This is concordant with previous research addressing other health-risk behaviours, including smoking cessation.^28,29^ The somewhat declining effect over time observed, again seen in other studies,^30–33^ suggests a potential advantage in the advice being repeated, allowing its effectiveness to be reinforced.

### Comparison with existing literature

Precaution with regards the sun is known to increase with age.^34–37^ Therefore, it is not surprising that a general increase of sun protection was seen in all three groups. Although age is the most likely explanation, it cannot be excluded that a general change of behaviour, on a society level, might also have played a role. Improved knowledge, repeated information campaigns, and other public health efforts may have contributed to enhanced awareness about UV exposure risks.^6^ Also, a general change of outdoor habits, especially among younger individuals reported to spend a declining proportion of time outdoors,^38,39^ may also have played a role. However, a predominant proportion of the study participants were aged >40 years at baseline, a stage at which increased caution in the sun particularly due to age would probably have already occurred.^34,35^ Finally, since all participants received some kind of intervention, it cannot be excluded that even the letter-mediated advice (Group 1) may have had an effect (which the authors failed to differentiate from ‘time‘), as previously found by Crane *et al*.^39^

In the extensive systematic reviews for the US Preventive Services Task Force,^9,40^ the role and effectiveness of PHC in mediating sun protection advice is underlined. Of the total 21 RCTs included in the 2018 review, however, only two studies^39,41^ followed their participants for more than 24 months. In both studies, the intervention was repeated at 12 and 24 months, with a final follow-up at 36 months. Prochaska *et al* followed the participants in two studies, based on individual stage-matched sun protection advice, for 24 months but, again, in both cases the intervention was repeated after 12 months.^42,43^ Thus, in no case was a longer follow-up period than 1 year after completed intervention used. In this regard, the present 10-year study’s results provide important, novel information. The fact that brief, personalised advice given by the GP induced a detectable effect as long as 10 years after delivery brings some contributory legitimacy to GPs spending part of their time and effort on personalised lifestyle counselling, as a natural component of the everyday patient work. Considering the relatively limited resource required (even if the intervention were to be repeated), the potential health economic benefits should not be underestimated; in fact, the cost-effectiveness of preventive efforts to increase sun protection, together with measures to enhance early skin cancer detection (such as regular self-examination), has been found to be substantial, both in terms of cost and quality-adjusted life-years, by reducing the burden of future advanced cancers.^3,4^

### Strengths and limitations

The most important strength of the study is its prospective design and long follow-up period. Although potentially successful, it is not possible to foresee whether an observed short-term behavioural change would be maintained over a sufficient period of time to result in any discernible effect on skin cancer risk. Therefore, these findings shed important light on how to design sustainable interventions to prevent skin cancer, using societal resources wisely. In this respect, the role and efficacy of healthcare providers in promoting favourable lifestyle habits, including those in PHC, is well documented.^42,44,45^ Since the study was performed in a true PHC setting, these results are likely to be appropriately transferrable to the PHC environment in general, as well as to similar healthcare settings, such as occupational health and dermatological clinics. Another strength of the study is its uncommonly high response rate, considering its long follow-up period, supporting the reliability of the results.

The study has some important limitations. Most importantly, it has a single-centre design, a circumstance to some extent limiting its generalisability. Also, selection bias due to attracting patients specifically interested in lifestyle matters cannot be excluded. If this occurred (a point of speculation), it may have reinforced the observed intervention effect. Another issue of importance to address is the clinical significance of the observed behavioural change found at 10 years, reflected in a reduction in SEPI score of on average 2 points, and its actual impact on morbidity and mortality. This is a point of speculation, and it is not possible to draw any conclusions from this study. The absence of a true control group, receiving no sun protection advice at all, may raise some criticism. However, the mere completion of a questionnaire mapping sun habits per se may be viewed as an intervention, for which reason this would not have guaranteed that no effect besides the effect of time or ageing would have had an impact on the outcome. With the present design, the letter group, containing the lowest level of intervention (arguably quite similar to normal practice in which information is often given as printed information added to a normal routine), might in this respect be considered as control group. The fact that a significantly greater increase in sun protection could be observed in the two doctor’s consultation groups than in the letter group consolidates an additional effect besides the likely effect of mere passage of time. Finally, it can be questioned whether the relatively high age distribution in the study population represents the most favourable target group for intervention. On the other hand, this age distribution is likely to be coherently representative of a general population of patients seeking PHC.

### Implications for research and practice

In conclusion, this study indicates that personalised sun protection advice mediated in person by the GP may lead not only to temporary, but also to long-term, persistent improvement of sun protective behaviour, albeit modest. The advice is likely to benefit from being repeated at appropriate intervals, for instance in association with nevi checks or other relevant medical consultations. Given the steadily increasing skin cancer incidence worldwide, these results indicate that sun protection advice should, by preference, be considered one of the natural areas of prevention to include in PHC patient consultations, along with other lifestyle directed advice probably more commonly addressed in practice. The possible interactions between sun protection advice and other behavioural counselling, and how these can be balanced in the case of contradictory advice (for instance, avoiding the sun versus performing outdoor physical activities), emerge as an area of potentially important interest for future research, which is to date sparsely explored. The actual impact and efficacy of repeated sun protection advice also needs further research.

## Trial registration

This trial was registered with the ISRCTN Registry (reference number: ISRCTN99837874)
